# Analysis of the Effect of Injuries on Match Performance Variables in Professional Soccer Players: A Retrospective, Experimental Longitudinal Design

**DOI:** 10.1186/s40798-022-00427-w

**Published:** 2022-03-03

**Authors:** Javier Raya-González, Juan José Pulido, Marco Beato, José Carlos Ponce-Bordón, Roberto López del Campo, Ricardo Resta, Tomás García-Calvo

**Affiliations:** 1 Faculty of Health Sciences, Universidad Isabel I, Burgos, Spain; 2 Faculty of Education and Psychology, University of Extremadura, Badajoz, Spain; 3 School of Health and Sports Science, University of Suffolk, Ipswich, UK; 4 Institute of Health and Wellbeing, University of Suffolk, Ipswich, UK; 5 Faculty of Sport Sciences, University of Extremadura, Cáceres, Spain; 6 LaLiga Sport Research Section, Madrid, Spain

**Keywords:** Physical conditioning, Human, External load, Playing time, Video tracking, Return to play, Football

## Abstract

**Background:**

Knowing the impact of injuries is essential for their adequate management during reconditioning programs.

**Objective:**

This study aimed to analyze the changes in match performance parameters in professional soccer players after sustaining an injury, which was defined according to injury severity.

**Methods:**

Two-hundred and seven injuries related to one hundred and sixty-one professional soccer players from the Spanish LaLiga™ were considered for this study. All the injuries were classified according to their severity as minor (from 4 to 7 missed days), moderate (from 8 to 28 missed days), and major (more than 28 missed days). Through Mediacoach^®^ videotracking system, time and external demand variables were collected and subsequently compared between pre-injury and return to play periods. The analyzed variables were (in m min^−1^): relative distance covered (RD; total distance covered·min^−1^), distance covered walking (0–6 km h^−1^), distance covered jogging (6–12 km h^−1)^, distance covered running (12–18 km h^−1^), distance covered at intense running (18–21 km h^−1^), distance covered at high-speed running (21–24 km h^−1^), and sprinting (> 24 km h^−1^) distance covered.

**Results:**

Significant reductions in playing time after suffering moderate and major injuries were observed. Significant reductions after minor injuries were observed in jogging (> 6 km h^−1^) and running (6–12 km h^−1^), while significantly greater distances at intense running (18–21 km h^−1^) and high-speed running (21–24 km h^−1^) were covered by players who suffer major injuries. Finally, relevant decreases in the maximum speed achieved after moderate and major injuries were found.

**Conclusions:**

In conclusion, this study shows the importance of high loads during reconditioning programs, as well as implementing strategies that allow reaching levels of maximum speed values after the return to play.

## Key Points

- Players who sustained moderate and major injuries had reduced playing time and participation.
- Players who suffered major injuries covered greater distances at intense running and high-speed running (21.0–24.0 km h^−1^) after the return to play.
- Decreases in the maximum speed achieved were found after moderate and major injuries.

## Introduction

Soccer is a highly demanding sport characterized by several high-intensity actions (e.g., jumps, sprints or change of directions) that players need to perform during their practice [1, 2]. Subsequently, soccer presents a great injury risk for players, who frequently suffer injuries during their career [3, 4]. In this regard, a recent meta-analysis [5] has revealed an overall incidence of injuries in professional male soccer players of 8.1 injuries/1000 h of exposure, with a higher injury incidence in matches (36 injuries/1000 h of exposure) than in training (3.7 injuries/1000 h of exposure). These injuries have a financial impact not only from the medical, surgical and rehabilitation perspective but also due to the high salaries that players receive during their absence period [6]. Likewise, injuries are associated with a reduction in team performance because of limited availability of players for competition [7]. In addition, injured players are at greater risk of reinjury [8] and to have long-term health consequences which influence their quality of life and career [9].

It is essential to consider that injuries involve a period of inactivity that leads to physical detraining [10, 11]. This process is defined as the partial or complete loss of training-induced anatomical, physiological and performance adaptations, as a consequence of training reduction or cessation [11]. The magnitude of the decrement is influenced by the inactivity duration [i.e., short term (< 4 weeks of insufficient training stimulus) or long-term detraining (> 4 weeks of insufficient training stimulus)] [11] and the athletes’ level, which is faster in elite athletes [12]. Specifically, with professional soccer players, recent studies have shown that detraining leads to a reduction of the maximal aerobic speed [13], an impairment of the maximal oxygen consumption [14], a blood hemoconcentration process [13], increases in the percentage of fat-mass [13, 15] and a decrement in lean-mass or fat-free mass [15]. These negative changes may affect the soccer players’ and team match performance during competition [16].

To minimize the effect of detraining in injured players, reconditioning programs have been developed and reported in the scientific literature [10, 17, 18]. These programs aim to facilitate a gradual and specific transition from the inactivity phase to the return to performance one [10], favoring a safe reincorporation to training and competition. Generally, reconditioning programs are based on individualized and decontextualized training tasks and injured players start to take part in regular soccer sessions in the last phases of the programs [10]. However, it has been previously shown that regular soccer training sessions do not replicate the demands of competition, being the match the most demanding session for players [1, 19, 20]. For this reason, players need to be exposed to a specific stimulus (i.e., simulated or friendly) matches in order to bridge the gap to competition and to get a fulfilled reconditioning process [17]. However, this is difficult in soccer because some external interests (e.g., television rights), as well as short-term performance results, play a role in training and match schedules [21]. In this regard, professional soccer players may compete (in some circumstances) after an injury without being fully prepared [22]. Therefore, it seems necessary to know the impact of injuries on match performance parameters to improve the efficacy of reconditioning programs for performance purposes as well as to design preventive programs to avoid reinjuries.

To date, only one study analyzed the impact of injuries on match performance parameters [23], which was focused mainly on hamstring injuries and all the participants belonged to the same team. Thus, further studies about this topic are warranted. Therefore, the aim of this study was to analyze the changes in match performance parameters (e.g., high speed running, maximum speed, playing time) in a large sample of professional soccer players after sustaining an injury, which was defined according to injury severity. We hypothesized that players’ match performance parameters are decreased in the first matches after the injury, but it improves to baseline level after certain number of matches.

## Methods

### Participants

Two hundred and seven injuries related to one hundred and sixty-one professional soccer players, who competed in the First Division of the Spanish Professional Football League (LaLiga™), composed the sample of this study. These injuries corresponded to 828 match observations (i.e., four matches pre-injury and four matches after the return to play for each selected injury). Injuries were selected for further analysis if the information about them was publicly provided by the soccer club and if the players participated at least in the aforementioned matches (i.e., four matches pre-injury and four matches after the return to play during at least 10 min). Goalkeepers were excluded from the analysis due to their specific role during matches. Data were provided by LaLiga™, who authorized the use of the variables included in this investigation. All data were anonymized according to the Declaration of HELSINKI to ensure players and teams confidentiality, and the protocol was fully approved by the Ethics Committee of the Universidad Isabel I (UI1-PI008).

### Procedures

A retrospective, descriptive longitudinal design was applied to analyze the influence of injuries on professional soccer players’ performance, in terms of participation time and external demands, after suffering an injury and differentiating among injury severity (i.e., minor, moderate and major). Injury data from official medical reports of seasons 2018/2019 and 2019/2020 were obtained, although injuries occurred during the post-COVID outbreak were not linked. This information was compared with the database provided by LaLiga™, in which information about playing time and external demands was facilitated. Those injuries that fitted the previously purposed inclusion criteria (i.e., player who suffer the injury participated in four matches pre-injury and four matches after the return to play during at least 10 min) were included in the statistical analysis. In this regard, mean values related to the four pre-injury matches were compared with the values obtained in each of the post-injury matches, differentiating between injuries according to their severity.

#### Injury Definitions

The UEFA guidelines for epidemiological studies were used in this study [24], so an injury was considered as a “situation that occurred during a scheduled training session or match that caused absence from the next training session or match”. Similarly, these guidelines were followed to differentiate the severity of injuries, classifying them into minor (4–7 days of absence), moderate (8–28 days of absence) and major (more than 28 days of absence), but slight injuries were excluded. For each injury, the following information was registered: injury date, return to play date, missed days, severity, missed matches and match typology after the injury.

#### Study Variables

*Time variables*—The playing time was recorded for each match. In addition, the participation in post-injury matches was registered dividing the players into starters (player who started the match) and non-starters (player who started the match on the bench). Also, starters were classified into those that played the entire match and those that were substituted, while non-starters were separated into those players that took part in the match during the first half and those players who took part in the match during the second half.

*External load variables*—The following variables were recorded in m min^−1^: relative distance covered (RD; total distance covered·min^−1^), distance covered walking (0–6 km h^−1^), distance covered jogging (6–12 km h^−1^), distance covered running (12–18 km h^−1^), distance covered at intense running (18–21 km h^−1^), distance covered at high-speed running (21–24 km h^−1^), and sprinting (> 24 km h^−1^) distance covered. These speed zones were used in previous soccer studies [25, 26]. Additionally, the maximum speed [peak speed (km h^−1^) reached by a soccer player in a match were registered [27]. All the study variables were collected through Mediacoach^®^ system, which is composed of a series of super 4 K-High Dynamic Range cameras based on a positioning system (Tracab—ChyronHego VTS). This system records from several angles and analyzes X and Y positions for each player, providing real-time three-dimensional tracking (acquisition frequency = 25 Hz). The validity and reliability of the Mediacoach^®^ system was previously reported by Rivilla-García et al. [28] and Pons et al. [26].

### Statistical analysis

Data were analyzed using SPSS v.25.0 for Windows (IBM, Armonk, NY USA). A linear mixed model (LMM) analysis was carried out for each of the eight variables using the MIXED procedure. LMM lets analyze data with a hierarchical structure in nesting units and has demonstrated its ability to cope with unbalanced and repeated-measures data [29]. For example, distance covered in matches are nested for players across time (i.e., each player has a record for any match played). To determine the need to use this statistical procedure, we first calculated the levels of within-person variance for each player by constructing unconditional null models. These unconditional models allowed to calculate the intraclass correlation coefficient (ICC), which showed values greater than 10%, which indicated the existence of variability in the data and justified this analysis approach [30]. Following that, some separate random intercept models were constructed for each outcome measure with matches and injury severity included as fixed effects. In this way, we compared the values of the variables that the players had in the 4 games before the injury, with each of the following 4 games after the injury. At the same time, the severity of the injuries and how they affected the next games were compared. Lastly, mean difference (Δ%) = (mean 1 − mean 2) × 100/mean 2) and Cohen’s effect sizes (ES) [31] were also calculated to quantify the magnitude of difference for all pairwise comparisons using the following thresholds for interpretation: trivial, < 0.20; small, 0.20–0.59; moderate, 0.60–1.19; large, 1.20–1.99; very large, 2.00–3.99; extremely large, > 4.00 [32].

## Results

A total of 207 injuries were recorded, which were classified as follow: 23 minor, 138 moderate and 46 major injuries. A mean of 1.45 ± 0.80 missed matches were observed when minor injuries were considered, while 2.50 ± 1.35, and 5.04 ± 2.25 missed matches were reported for moderate and major injuries, respectively. Additionally, minor injuries entailed 5.95 ± 1.05 missed days, moderate injuries 17.93 ± 6.37 missed days and major injuries 42.09 ± 14.36 missed days.

Participation typology (i.e., starter or non-starter) in post-injury matches attending to injury severity is recorded in Table 1. Participation of the players as starters increased throughout the matches following any type of injury—minor injuries were those with the highest values in this category. Instead, players who suffered major injuries presented the lowest values in starter participation in all matches, although similar values were observed for moderate injuries. Consequently, players who suffered moderate and major injuries reported higher values in substituted and in non-starter conditions after return to play, although these values were reduced throughout the matches. Finally, in eight cases of moderate and major injuries, players participated as starters, but they were substituted during the first half of the match.

**Table 1:** Participation typology in post-injury matches by injury severity

Injuries	Match typology															
	+ 1				+ 2				+ 3				+ 4			
	ST		N-ST		ST		N-ST		ST		N-ST		ST		N-ST	
	C Number (%)	S Number (%)	FH Number (%)	SH Number (%)	C Number (%)	S Number (%)	FH Number (%)	SH Number (%)	C Number (%)	S Number (%)	FH Number (%)	SH Number (%)	C Number (%)	S Number (%)	FH Number (%)	SH Number (%)
Minor (*n* = 23)	13 (56.5)	2 (8.7)	0 (0.0)	8 (37.8)	17 (73.9)	4 (17.4)	0 (0.0)	2 (8.7)	19 (82.6)	3 (13.0)	0 (0.0)	1 (4.4)	16 (69.6)	4 (17.4)	0 (0.0)	3 (13.0)
Moderate (*n* = 138)	69 (50.0)	14 (10.1)	3 (2.2)	52 (37.7)	73 (52.9)	37 (26.8)	0 (0.0)	28 (20.3)	82 (59.4)	27 (19.6)	1 (0.7)	28 (20.3)	78 (56.5)	33 (23.9)	1 (0.7)	26 (18.9)
Major (*n* = 46)	15 (32.6)	5 (10.9)	3 (6.5)	23 (50.0)	23 (50.0)	16 (34.8)	1 (2.2)	6 (13.0)	30 (65.4)	4 (8.7)	0 (0.0)	12 (23.9)	22 (47.8)	14 (30.4)	0 (0.0)	10 (21.8)

*ST* starter, *N-ST* non-starter, *C* match played entirely, *S* substituted during the match, *FH* first half, *SH* second half

Table 2 shows the differences in the time played during matches in relation to the injury severity. No significant differences in playing time were reported in minor injuries in any post-injury match compared to pre-injury values, while in moderate injuries significant differences were reported when pre-injury values were compared with + 1 (*p* = 0.000, ES =  − 9.20), + 2 (*p* = 0.008, ES =  − 3.67), and + 4 (*p* = 0.02, ES =  − 3.26) matches. In major injuries, significant differences were revealed in + 1 (*p* = 0.000, ES =  − 10.69), and + 4 (*p* = 0.03, ES =  − 3.26) matches. Additionally, a greater effect was observed in moderate and major injuries in + 3 matches compared to minor injuries, while major injuries reported a higher effect in + 1 matches in comparison to minor and moderate ones.

**Table 2:** Differences in the minutes played during matches by injury severity

Injuries	Match typology					Pair comparisons (mean differences %; ES)			
	Pre-injury (Mean ± SD)	+ 1 (Mean ± SD)	+ 2 (Mean ± SD)	+ 3 (Mean ± SD)	+ 4 (Mean ± SD)	Pre versus + 1	Pre versus + 2	Pre versus + 3	Pre versus + 4
Minor	78.00 ± 3.57	68.60 ± 5.45	84.10 ± 5.54	88.90 ± 5.54	83.30 ± 5.54	− 12.05; − 2.63	7.82; 1.71	13.97; 3.05	6.79; 1.48
Moderate	80.04 ± 1.70	64.40 ± 2.39	73.80 ± 2.39	75.90 ± 2.38^a^	74.50 ± 2.39	− 19.54; − 9.20***	− 7.80; − 3.67**	− 5.17; − 2.44	− 6.92; − 3.26*
Major	82.50 ± 2.76	53.00 ± 4.00^ab^	77.50 ± 4.04	74.30 ± 4.04^a^	73.50 ± 4.04	− 35.76; − 10.69***	− 6.06; − 1.81	− 9.94; − 2.97	− 10.91; − 3.26*

*ES* effect size, *SD* standard deviation, *pre* mean of the last four matches before injury, + *1* first match after return to play, + *2* s match after return to play, + *3* third match after return to play, + *4* fourth match after return to play

*Significant level was set at *p* < 0.05; **Significant level was set at *p* < 0.01; ***Significant level was set at *p* < 0.001

^a^Significant greater effect compared to minor injuries, ^b^Significant greater effect compared to moderate injuries

Differences in distances covered at low and medium intensities by soccer players between pre-and post-injury matches in relation to the injury severity are reported in Table 3. Following minor injuries, differences with pre-injury values were found in the distance covered jogging (+ 1 match; *p* = 0.03, ES =  − 2.24), and in the distance covered running (+ 2 match; *p* = 0.004, ES =  − 3.77). Following moderate injuries, differences with pre-injury values were reported in the distance covered running (+ 1 match; *p* = 0.001 ES = 2.94), while significant differences were observed for major injuries in this last category (+ 4 match; *p* = 0.04, ES = 2.21). Additionally, between-severities analyses reported differences between moderate and minor injuries in RD (+ 1 match) and in running variable (in all the matches), as well as significant differences were found between major and minor injuries in RD (+ 3 match), jogging (+ 2 and + 3 matches), and running (+ 2, + 3, + 4 matches) variables. Finally, significant differences between moderate and major injuries were observed in the distance covered at jogging speed (+ 2 match).

**Table 3:** Differences in distances covered at low and medium intensities by soccer players between pre-and post-injury matches by injury severity

Physical responses	Injuries	Match typology					Pair comparisons (mean differences %; ES)			
		Pre-injury (Mean ± SD)	+ 1 (Mean ± SD)	+ 2 (Mean ± SD)	+ 3 (Mean ± SD)	+ 4 (Mean ± SD)	Pre versus + 1	Pre versus + 2	Pre versus + 3	Pre versus + 4
RD (m min^−1^)	Minor	110 ± 1.28	107 ± 1.82	107 ± 1.84	107 ± 1.84	107 ± 1.87	− 2.72; − 2.34	− 2.72; − 2.34	− 2.72; − 2.34	− 2.72; − 2.34
	Moderate	109 ± 0.76	111 ± 0.92^a^	109 ± 0.92	109 ± 0.92	109 ± 0.92	1.83; 2.63	–	–	–
	Major	111 ± 1.08	112 ± 1.14	113 ± 1.42	113 ± 1.42^a^	113 ± 1.42	0.90; 0.93	1.80; 1.85	1.80; 1.85	1.80; 1.85
Walking (m min^−1^)	Minor	12.10 ± 0.28	12.60 ± 0.39	12.40 ± 0.39	12.10 ± 0.39	12.30 ± 0.40	4.13; 1.79	2.48; 1.07	–	1.65; 0.71
	Moderate	12.80 ± 0.15	12.90 ± 0.19	12.80 ± 0.19	13.00 ± 0.19	12.60 ± 0.19	0.78; 0.67	–	1.56; 1.33	− 1.56; − 1.33
	Major	12.70 ± 0.23	12.60 ± 0.30	12.60 ± 0.30	12.60 ± 0.30	12.30 ± 0.30	− 0.79; − 0.43	− 0.79; − 0.43	− 0.79; − 0.43	− 3.15; − 1.74
Jogging (m min^−1^)	Minor	38.40 ± 0.67	36.30 ± 0.95	36.90 ± 0.96	36.70 ± 0.96	37.20 ± 0.97	− 5.47; − 3.13*	− 3.9; − 2.24	− 4.43; − 2.54	− 3.13; − 1.79
	Moderate	38.40 ± 0.40	38.30 ± 0.48	37.90 ± 0.48	38.00 ± 0.48	38.00 ± 0.48	− 0.26; − 0.25	− 1.3; − 1.25	− 1.04; − 1.00	− 1.04; − 1.00
	Major	39.10 ± 0.56	38.50 ± 0.74	40.40 ± 0.74^ab^	39.80 ± 0.74^a^	38.40 ± 0.74	− 1.53; − 1.07	3.32; 2.32	1.79; 1.25	− 1.79; − 1.25
Running (m min^−1^)	Minor	29.70 ± 0.77	27.90 ± 1.06	26.80 ± 1.07	28.10 ± 1.07	27.80 ± 1.09	− 6.06; 0.26	− 9.76; − 3.77**	− 5.39; − 2.08	− 6.40; − 2.47
	Moderate	28.60 ± 0.51	30.10 ± 0.59^a^	28.80 ± 0.59^a^	29.30 ± 0.59^a^	29.90 ± 0.48^a^	5.24; 2.94**	0.70; 0.39	2.45; 1.37	4.55; 2.55
	Major	29.70 ± 0.68	30.30 ± 0.85	30.30 ± 0.85^a^	30.60 ± 0.85^a^	31.20 ± 0.85^a^	2.02; 0.88	2.02; 0.88	3.03; 1.32	5.05; 2.21*

*SD* standard deviation, + *1* first match after return to play, + *2* s match after return to play, + *3* third match after return to play, + *4* fourth match after return to play, *RD* relative distance; walking: distance covered below of 6.0 km h^−1^; jogging: distance covered between 6.0 and 12.0 km h^−1^; running: distance covered between 12.0 and 18.0 km h^−1^

*Significant level was set at *p* < 0.05, **Significant level was set at *p* < 0.01, ***Significant level was set at p < 0.001

^a^Significant greater effect compared to minor injuries, ^b^Significant greater effect compared to moderate injuries

In Table 4 are presented the differences in distances covered at high intensities by soccer players between pre-and post-injury matches in relation to the injury severity. Following minor injuries, significant differences were observed in the distance sprinting (+ 2 match; *p* = 0.04, ES = 3.05) but not in the other metrics. Moderate injuries presented significant differences with pre-injury values in maximum speed (+ 1, + 2 and + 4 matches; *p* = 0.001–0.000, ES =  − 5.38 to − 3.85), while following major injuries significant differences were found in intense running (+ 1 and + 4 matches; *p* = 0.04, ES = 2.41) and high-speed running (+ 4 match; *p* = 0.04, ES = 2.60) variables, as well as in maximum speed (+ 1, + 2 and + 3 matches; *p* = 0.03–0.000, ES =  − 6.00 to − 5.38). In addition, between-severity analyses found differences between moderate and minor injuries in maximum speed (+ 2 and + 4 matches), as well as differences between major and minor injuries were found in intense running (+ 3 and + 4 matches), high speed running (+ 4 match), and maximum speed (+ 2, + 3 and + 4 matches). Finally, only significant differences between moderate and major injuries were reported in maximum speed (+ 3 match).

**Table 4:** Differences in distances covered at high intensities by soccer players between pre-and post-injury matches by injury severity

Physical responses	Injuries	Match typology					Pair Comparisons (Mean Differences %; ES)			
		Pre-injury (Mean ± SD)	+ 1 (Mean ± SD)	+ 2 (Mean ± SD)	+ 3 (Mean ± SD)	+ 4 (Mean ± SD)	Pre versus + 1	Pre versus + 2	Pre versus + 3	Pre versus + 4
Intense running (m min^−1^)	Minor	6.82 ± 0.26	6.62 ± 03.38	6.69 ± 0.38	6.34 ± 0.38	6.23 ± 0.39	− 2.93; − 0.77	− 1.91; − 0.5	− 7.04; − 1.85	− 8.65; − 2.27
	Moderate	6.70 ± 0.15	6.99 ± 0.19	6.73 ± 0.19	6.63 ± 0.19	6.89 ± 0.19	4.33; 1.93	0.45; 0.20	− 1.04; − 0.47	2.84; 1.27
	Major	6.84 ± 0.22	7.37 ± 0.29	6.78 ± 0.29	7.28 ± 0.29^a^	7.37 ± 0.29^a^	7.75; 2.41*	− 0.88; − 0.27	6.43; 2.00	7.75; 2.41*
High-speed running (m min^−1^)	Minor	3.72 ± 0.18	3.64 ± 0.27	3.55 ± 0.27	3.56 ± 0.27	3.40 ± 0.28	− 2.15; − 0.44	− 4.57; − 0.94	− 4.30; − 0.89	− 8.60; − 1.78
	Moderate	3.73 ± 0.10	3.87 ± 0.13	3.77 ± 0.13	3.78 ± 0.13	3.76 ± 0.13	3.75; 1.40	1.07; 0.40	1.34; 0.50	0.80; 0.30
	Major	3.70 ± 0.15	3.80 ± 0.20	3.80 ± 0.21	3.98 ± 0.21	4.09 ± 0.21^a^	2.70; 0.67	2.70; 0.67	7.57; 1.87	10.54; 2.60*
Sprinting (m min^−1^)	Minor	2.79 ± 0.22	2.81 ± 0.32	3.46 ± 0.32	2.84 ± 0.32	2.70 ± 0.33	0.72; 0.09	24.01; 3.05*	1.79; 0.23	− 3.23; − 0.41
	Moderate	2.90 ± 0.12	2.84 ± 0.15	2.90 ± 0.15	2.95 ± 0.15	2.76 ± 0.15	− 2.07; − 0.50	–	1.72; 0.23	− 4.83; − 1.17
	Major	2.81 ± 0.18	3.19 ± 0.24	2.72 ± 0.24	2.91 ± 0.24	2.95 ± 0.24	13.52; 2.11	− 3.20; − 0.50	3.56; 0.56	4.98; 0.78
Maximum speed (km h^−1^)	Minor	29.90 ± 0.25	29.50 ± 0.38	30.60 ± 0.38	30.50 ± 0.38	30.50 ± 0.39	− 1.34; − 1.60	2.34; 2.80	2.01; 2.40	2.01; 2.40
	Moderate	30.10 ± 0.13	29.40 ± 0.17	29.50 ± 0.17^a^	29.90 ± 0.17	29.60 ± 0.17^a^	− 2.33; − 5.38***	− 1.99; − 4.61***	− 0.66; − 1.54	− 1.66; − 3.85**
	Major	30.30 ± 0.20	29.10 ± 0.28	29.60 ± 0.28^a^	29.20 ± 0.28^ab^	29.7 ± 0.28^a^	− 3.96; − 6.00***	− 2.31; − 5.38*	− 3.63; − 5.5***	− 1.98; − 3.00

*SD* standard deviation, + *1* first match after return to play, + *2* s match after return to play, + *3* third match after return to play, + *4* fourth match after return to play; intense running: distance covered between 18.0 and 21.0 km h^−1^; high speed running: distance covered between 21.0 and 24.0 km h^−1^; sprinting: distance covered above 24.0 km h^−1^

*Significant level was set at *p* < 0.05, **Significant level was set at *p* < 0.01, ***Significant level was set ^a^t *p* < 0.001

^a^Significant greater effect compared to minor injuries, ^b^Significant greater effect compared to moderate injuries

## Discussion

The aim of this study was to analyze the changes in match performance parameters (e.g., high speed running, maximum speed, playing time) in professional soccer players after sustaining an injury, adjusting for injury severity. The main novelty of this study is the pre-post injury comparison of external loads combined with playing time in professional soccer players. This study showed a significant reduction in playing time after suffering moderate and major injuries, because players were less used as starters following major injuries after the return to play. Additionally, significant reductions were observed in distances covered in jogging and running speed after minor injuries, while players who suffer major injuries covered significantly greater distances at intense running and high-speed running (mainly in + 4 match). Finally, a decrement in the maximum speed was found after moderate and major injuries. In this sense, the initial hypothesis has been verified.

To fully understand the real impact of injuries, it is necessary to evaluate performance variables and playing time in post-injury matches. This investigation analyzed the participation of injured players after the return to play, showing that players returned to be used as starters with the progressive succession of the matches considering every injury severity. Participation as starters after a major injury in early matches after the return to play (i.e., + 1 and + 2 matches) was reduced. This could be due to the necessity of a progressive return to competition when the amount of absence was longer, which is due to the severity of the injury [10, 33], which influenced the overall playing time. Although a reduction in playing time was not found after minor injuries, the results of the present study showed that this is the case after suffering moderate or severe injuries (mainly in + 1 matches). This finding represents a key problem for the team performance because the athletes’ availability following a moderate or major injury was prolonged and went beyond the return to play [10, 34]. Team performance could have been affected because specific players did not perform similar match performance parameters as before injury (such as minutes of play) until they have played some competitive matches that were needed to recover the previous physical level [7, 35].

Common parameters in the analysis of external demands in soccer matches are the total distance (TD) and RD [36], so it seems pertinent to analyze the impact of injuries on these variables after the return to play. In this sense, no significant differences in RD between the pre-and post-injury values were reported for any injury severity. However, while a trend toward a reduction in RD was observed after minor injuries, an increasing trend was found after moderate and major injuries, which could have been possibly influenced by the reduction in playing time experienced by players who suffered these injuries. On the other hand, although no significant differences were observed in walking distance in any severity, players who suffered minor injuries revealed a significant reduction in the distance covered at jogging and running during first matches, showing significant differences with moderate and major injuries. We suppose that players suffering minor injuries could have participated in official matches without being fully recovered in terms of health readiness [21], which could have altered their match performance (this is a possible explanation). According to a previous study [23], we have found a significant post-injury increment in the running speed distance in players suffering moderate and major injuries, which were significantly higher compared with minor injuries.

Since the positive influence of high-intensity actions on soccer players’ performance during matches [27, 37], the monitoring and control of these actions after an injury seem crucial to ensure an adequate return to play [33]. Players who suffered major injuries reported significantly greater distances in the intense running and high-speed running categories, mainly in the last match analyzed (i.e., + 4), which is in agreement with previous research [23]. Some motivations of this result could be associated with the fact that these players have dedicated more time to train high-speed running [33], along with greater control of the load and fatigue during the reconditioning program [23], or, another possible explanation is that players suffering minor injuries were reincluded in the team as starters before a full recovery, therefore their match performance was affected. These findings show that after sustaining moderate and major injuries, soccer players returned to play executing similar or even better external performance compared to their previous level, however authors cannot explain the motivations of this with certainty. The external load metrics reported in this study were unified based on players played time, therefore the reduced playing time reported after some injuries can explain the similar external load performance reported compared to pre-injury. Although players maintained their pre-injury performance, they did it for less time, especially in the + 1 match. For this reason, it seems appropriate to suggest that during rehab programs external demands should not only be controlled in terms of absolute values (volume = 45 or 90 min), but also in relation to time (intensity = per min). Practitioners should develop return to play protocols that have a similar relative intensity of the matches and, with the right progression, increase the overall volume (training time) to fully prepare their players for the match.

Regarding the distance covered at sprinting speed, only certain increases were observed after minor injuries, while these were not reported after moderate and major injuries. This seems to be related to the maximum speed variable, which is significantly impaired after returning to play after suffering moderate and major injuries along the four matches analyzed. These results do not support those previously reported by Jiménez-Rubio et al. [23], who observed significant improvements in the maximum speed after the return to play compared with previous values. These results, which are in contrast with ours, could be due to methodological differences between the studies since in the aforementioned investigation all the players followed the same reconditioning program after they sustained the same injury (i.e., grade IIb hamstring strain injury), while a smaller sample was used (22 vs. 207 injuries). This reduction in the maximum speed observed in our study may suggest that soccer players, who have sustained moderate and major injuries, were not as physically fit as before the injuries despite returning to play protocols. Thus, they needed several official matches to regain their previous level, which may have a negative effect on the team performance given the importance of maximum speed during the match [37]. Therefore, strategies to improve the maximum speed level should be implemented during reconditioning processes.

This study is not without its limitations, firstly, only time parameters and external demands were considered, despite the importance of physiological variables during return to sport protocols [38, 39]. Secondly, this study focused on external load parameter recorded during matches, while physical tests may have added some important information. Furthermore, only players from the Spanish Premier League were involved in this study, and this could limit the application of these findings to other leagues. This is due to the fact that competitive demands vary between the main professional soccer leagues, however, future studies could replicate the protocol reported in this study and investigate other championships. Finally, there is no information on the rehabilitation programs followed by each team, which could have been different among them, and therefore, they may have influenced the post-injury performance reported in this study.

## Conclusions

This study compared the pre-post injury external loads combined with playing time in professional soccer players, which showed a reduction in playing time and in the participation as starters after moderate and major injuries. Moreover, a decrement in the distance covered at jogging and running speed was found in the first matches after minor injuries, as well as an increment in intense running and high-speed running after some matches (mainly in + 4 match) was reported after a major injury. Finally, it was observed a reduction in maximum speed in players suffering moderate and major injuries, which can suggest the necessity of a specific focus on sprinting and peak speed training in the final part of the rehabilitation programs (after these injuries) in order to prepare the players for the intensity required during competitions.

## Practical Applications

Authors suggest to practitioners that soccer players who have suffered minor injuries return to the competition safely and without haste, to avoid the risk of new injuries or re-injuries. This is also supported by the fact that players’ match performance after a minor injury remains similar to pre-injury level. Although no reduction/improvement in external load performance, it is pertinent to highlight that this is true for relative variables (m min^−1^), however, players maintain their performance but for a lower amount of time than during their pre-injury matches. In this sense, it seems necessary to periodize the rehabilitation programs to reproduce competitive demands considering both game intensity and volume. Finally, because of the lower maximum speed reported after moderate and major injuries, practitioners should implement strategies that allow players to reach their pre-injury speed levels in the rehabilitation processes.

## Data Availability

Restrictions apply to the availability of these data. Data were obtained from LaLiga and are available at https://www.laliga.es/en with the permission of LaLiga.

## Abbreviations

ICC: Intraclass correlation coefficient
ES: Cohen’s effect size
LMM: Linear mixed model
Δ%: Mean difference
RD: Total distance covered·min^−1^
