# Correction: Analysis of the Effect of Injuries on Match Performance Variables in Professional Soccer Players: A Retrospective, Experimental Longitudinal Design

**DOI:** 10.1186/s40798-022-00459-2

**Published:** 2022-05-01

**Authors:** Javier Raya-González, Juan José Pulido, Marco Beato, José Carlos Ponce-Bordón, Roberto López del Campo, Ricardo Resta, Tomás García-Calvo

**Affiliations:** 1 Faculty of Health Sciences, Universidad Isabel I, Burgos, Spain; 2 Faculty of Education and Psychology, University of Extremadura, Badajoz, Spain; 3 School of Health and Sports Science, University of Suffolk, Ipswich, UK; 4 Institute of Health and Wellbeing, University of Suffolk, Ipswich, UK; 5 Faculty of Sport Sciences, University of Extremadura, Cáceres, Spain; 6 LaLiga Sport Research Section, Madrid, Spain

## Correction: Sports Medicine - Open (2022) 8:31 10.1186/s40798-022-00427-w

The original article contained an error in attribution of affiliation to co-author, Ricardo Resta which has since been corrected.

